# Isoprostanoids in Clinical and Experimental Neurological Disease Models

**DOI:** 10.3390/antiox7070088

**Published:** 2018-07-11

**Authors:** Cinzia Signorini, Claudio De Felice, Jean-Marie Galano, Camille Oger, Silvia Leoncini, Alessio Cortelazzo, Lucia Ciccoli, Thierry Durand, Joussef Hayek, Jetty Chung-Yung Lee

**Affiliations:** 1 Department of Molecular and Developmental Medicine, University of Siena, I-53100 Siena, Italy; ciccolil@yahoo.it; 2 Neonatal Intensive Care Unit, Azienda Ospedaliera Universitaria Senese, I-53100 Siena, Italy; geniente@gmail.com; 3 Institut des Biomolécules Max Mousseron (IBMM), UMR 5247, Université de Montpellier, CNRS, ENSCM, F-34093 Montpellier CEDEX 05, France; jean-marie.galano@umontpellier.fr (J.-M.G.); camille.oger@umontpellier.fr (C.O.); thierry.durand@umontpellier.fr (T.D.); 4 Child Neuropsychiatry Unit, Azienda Ospedaliera Universitaria Senese, I-53100 Siena, Italy; s.leoncini74@gmail.com (S.L.); corteale@gmail.com (A.C.); j.hayek@ao-siena.toscana.it (J.H.); 5 School of Biological Sciences, The University of Hong Kong, Hong Kong, China; jettylee@hku.hk

**Keywords:** isoprostanes, neuroprostanes, neurological diseases, biomarkers

## Abstract

Isoprostanoids are a large family of compounds derived from non-enzymatic oxidation of polyunsaturated fatty acids (PUFAs). Unlike other oxidative stress biomarkers, they provide unique information on the precursor of the targeted PUFA. Although they were discovered about a quarter of century ago, the knowledge on the role of key isoprostanoids in the pathogenesis of experimental and human disease models remains limited. This is mainly due to the limited availability of highly purified molecules to be used as a reference standard in the identification of biological samples. The accurate knowledge on their biological relevance is the critical step that could be translated from some mere technical/industrial advances into a reliable biological disease marker which is helpful in deciphering the oxidative stress puzzle related to neurological disorders. Recent research indicates the value of isoprostanoids in predicting the clinical presentation and evolution of the neurological diseases. This review focuses on the relevance of isoprostanoids as mediators and potential biomarkers in neurological diseases, a heterogeneous family ranging from rare brain diseases to major health conditions that could have worldwide socioeconomic impact in the health sector. The current challenge is to identify the preferential biochemical pathways that actually follow the oxidative reactions in the neurological diseases and the consequence of the specific isoprostanes in the underlying pathogenic mechanisms.

## 1. Introduction

Over the years, lipid peroxidation went from being a mere indicator of oxidative stress process in in vitro experimental models to become a reliable marker of oxidative damage in vivo. One major part of lipid peroxidation is the non-enzymatic/free radical process triggered by the reaction of lipids with molecular oxygen, creating initial oxidative event that is further amplified through a series of chain reactions. The chemistry of the free radical–induced lipid peroxidation chain has been thoroughly reviewed by Yin et al. [1]. Decomposed oxidized lipids (i.e., aldehydes) [2] or non-enzymatic oxygenated metabolites (i.e., isoprostanoids) are known as the secondary products of lipid peroxidation [3]. Isoprostanoids, which are prostaglandin isomers, are derived from polyunsaturated fatty acids (PUFAs). Unlike enzymatically produced prostaglandins, isoprostanoids are formed in situ, within the membrane, and released through hydrolysis via phospholipase A_2_ (PLA_2_) [4].

Despite the growing interest in the product of protein gene expressions and molecular biology techniques, that are related to the complex signal transduction process, scientists should bear in mind that lipids are core components of the cell membrane in maintaining cellular structure and function.

Among the omics tools including genomics, transcriptomics, and metabolomics, lipidomics is prevalent in elucidating the pathogenesis of human diseases associated to lipids [5] and also in the development of precision medicine, which requires molecular diagnostic tests. In retrospect, the contradiction to lipidomics of isoprostanoids is influenced by cyclooxygenase-2 (COX-2) reaction products [5]. Nevertheless, isoprostanoids are investigated to identify the biological involvement and its role in the pathogenic pathways.

## 2. Relevance for Lipids in Brain

Lipids, in particular phospholipids, are involved in maintaining the functionality of neuronal cell membrane where the synaptic transmission depends on. For instance, synaptic phospholipids have been suggested as a new target for cortical hyperexcitability in psychiatric disorders [6], and synaptic lipid signaling has been shown to be involved in the glutamatergic transmission in the somatosensory cortex [7], where an alteration has been evoked in the pathophysiology of psychiatric disorder [8]. In the aging brains of healthy individuals, an association between changes in neuronal electrical excitability and the oxidation of membrane lipids appears to be related in the decline of learning and memory performance. A hypothetical explanation would lie in the fact that processes triggered by free radical and oxidant reactions on the lipid moiety would contribute to the age-related deterioration of the nervous system by damaging the phospholipids of the cell and organelle membranes. Since the peroxidized portion of the PUFAs are excised from the phospholipid by PLA_2_ enzymes, it has been proposed that the balance between PLA_2_ and redox status would determine the rate of lipid peroxidation in the membrane and perhaps affect and deteriorate the PLA_2_-dependent neuronal excitability and plasticity [9].

When searching for a biomarker related to diseases, the general trend is to attempt to identify a specific protein even though the lipid portion can be equally agreeable for the identification, especially in neurological diseases due to the high lipid content in the brain. In this regard, the relevance of lipidomics as a novel method to identify biomarkers in early detection and diagnostic criteria for Alzheimer’s disease was recently reviewed [10], and furthermore, mass spectrometry–derived lipidomic profiles appeared to be different in individuals with and without cognitive impairment [11]. Therefore, complete or targeted lipidomics appears to be a relevant approach in search of biomarkers, but it is relatively unexplored when compared to the large clinical application for proteomics.

**Figure 1:**
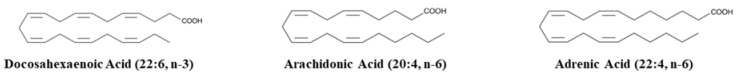
Chemical structures of key polyunsaturated fatty acids related to neuronal phospholipids.

In lipid metabolism, dietary essential n-3 and n-6 polyunsaturated fatty acids (PUFAs), namely α-linolenic acid (ALA, 18:3n-3) and linoleic acid (LA, 18:2n-6), play crucial roles in maintaining tissue levels [12]. However, docosahexaenoic acid (DHA; 22:6, n-3), and arachidonic acid (AA; 20:4, n-6), from the diet are more important PUFAs as they are abundant in the brain and vital components of the neuronal phospholipids. Further, in n-6 PUFA metabolism, adrenic acid (AdA; 22:4, n-6) is produced by elongation of AA and concentrated in the myelin sheath within the brain white matter of the primates. PUFAs are esterified in situ to phospholipids and in particular, DHA, AA, and AdA (Figure 1) are crucial components of neuronal or glia phospholipids.

PUFAs are related to the physiological developmental of the neurological system in the brain when considering the pathogenesis related to neuroplasticity, neurogenesis, and synaptogenesis [13]. In relation, nutritional n-3 PUFAs deficiency, especially during the perinatal period, alters neuronal plasticity [14]. Moreover, it is suggested that a good balance of n-3/n-6 PUFA ratio protects the cognitive deficits induced by neuroinflammation [15], and furthermore enrichment of n-3 PUFAs mediate mechanisms involved in learning memory performance [16].

As shown in the brain of rodents, DHA and AA distribution is region–specific and needed for brain function and development [17]. In particular, DHA promotes neuroplasticity, neurogenesis, synaptogenesis and neuroimmune interactions [18,19]. In a recent report, enriched brain levels of DHA by genetic conversion of n-6 PUFA to n-3-PUFA led to increased hippocampal neurogenesis [20]. Neurogenesis was also increased in the hippocampus of aged rats supplemented with DHA [19]. On the other hand, Coti Bertrand et al., demonstrated that neurogenesis was decreased in brain of DHA-deficient embryonic rat [21].

## 3. Relevance of Lipid Peroxidation Products in Neurodegeneration

In brain, lipid peroxidation products, namely malondialdehyde (MDA), protein-bound acrolein adduct, and isoprostanoids, are reported to be elevated in the progression of Alzheimer’s disease (AD) from the earliest to the late stages and were also detected in mild cognitive impairment (MCI) conditions [22]. As a result, these products are currently suggested to contribute to neurodegeneration. In addition, immunochemical detection of lipid hydroperoxide- and aldehyde-modified proteins, and selective protein targets of aldehydes were found in AD [23,24]. Nevertheless, relationships between lipid peroxidation products and key clinical AD features remain to be confirmed.

**Figure 2:**
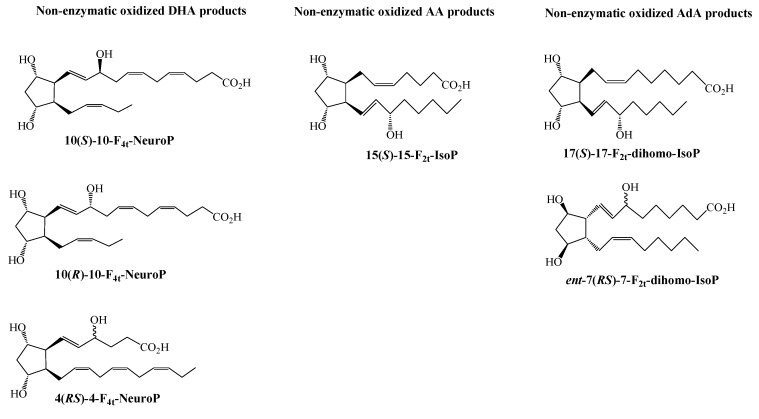
Chemical structures related to oxidized products of docosahexaenoic acid (DHA), arachidonic acid (AA), and adrenic acid (AdA). Legend: IsoP: isoprostane; NeuroP: neuroprostane.

Of note in this review is the uniqueness of isoprostanoids in neurodegeneration. Through interaction of radical species, such as radical oxygen species (ROS), PUFAs undergo a series of non-enzymatic lipid peroxidation and generate specialized lipid mediators such as F_2_-isoprostanes (F_2_-IsoPs), F_2_-dihomo-isoprostanes (F_2_-dihomo-IsoPs), and F_4_-neuroprostanes (F_4_-NeuroPs) from AA, AdA, and DHA, respectively. Among all the isoprostanoids identified so far, the chemical structures related closest to neurological diseases [25,26,27,28] are reported in Figure 2.

The result of cyclization process in AA oxidation forms four series comprising of 5-, 8-, 12-, and 15-F_2_-Isoprostanes (IsoP). F_2_-IsoPs have been defined as the gold standard marker of lipid peroxidation in vivo for their relevance in several human diseases, including neurodegenerative disease [29,30].

Moreover, AdA oxidation form four series of regioisomers (7-, 10-, 14-, and 17-series) [31], and were detected in AD patients [31,32], and also in Rett syndrome (RTT) [25], Down syndrome [33], and epileptic [34] patients. The myelin sheath is concentrated with AdA and F_2_-dihomo-IsoPs have been suggested to be specific in assessing the extent of free radical damage of the myelin [31]. Likewise, F_4_-NeuroPs measurements are relatively specific to oxidative damage of the neuronal membranes due to abundant DHA in the brain gray matter [35,36]. In this regard, F_4_-NeuroPs, and not F_2_-IsoPs, were proposed to be the critical group of oxidized DHA products for neuronal damage [37,38,39]. F_2_-IsoPs and F_4_-NeuroPs were also investigated as biomarkers to explore the role of the oxidative damage in the pathogenesis of Parkinson’s disease [28]. In such neurological conditions, plasma levels of F_2_-IsoPs and F_4_-NeuroPs provided evidence that peripheral indices of oxidative damage are elevated at different stages of the disease [28].

Non-enzymatic DHA oxidation generates eight regioisomer series (4-, 7-, 10-, 11-, 13-, 14-, 17- or 20-series). Among all the F_4_-NeuroP molecules, a few isomers were characterized in neuro-pathological conditions, and so far, 4-F_4t_-NeuroPs and 10-F_4t_-NeuroPs are the most represented [3,27,34,40]. Since 1999, F_4_-NeuroPs have been investigated as specific marker of DHA peroxidation in AD [41], and the interest in such matter is continuing [42]. Notably, the debate on the use of nutraceutical intervention specifically, DHA supplementation is ongoing. In this regard, PUFAs and DHA levels are reported to be associated to reduced risk of AD. Nevertheless, lipid peroxidation products of n-3-PUFA have been associated to increased levels of β-amyloid peptide in in vitro studies [43], indicating that biological activities are stimulated by oxidized DHA and should be asserted in nutrition related studies. Considering this, caution must also be made in scientific experimentations as PUFAs are prone to oxidation in in vitro, whereas supplementation of PUFA reduce in vivo oxidative stress by lowering F_2_-IsoPs levels [44,45]; the two outcomes may not be complementary.

Overall, PUFAs and their oxygenated metabolites of non-enzymatic lipid peroxidation have been investigated as potential biomarkers and/or therapeutic target in different neurological diseases. The measurement of F_2_-IsoPs can be accurately carried out in all body fluids by mass spectrometric analyses [46,47]. Currently, such detections are performed to evaluate the occurrence of lipid peroxidation events in numerous diseases including neurodegenerative diseases [48,49,50]. Nevertheless, it has been focused on the possibility that single F_2_-IsoPs measurements could represent “spot” measurements of lipid peroxidation process rather than understanding the real biological role of the isoprostanoids [51]. As a biomarker, detection of different isomers and/or metabolites [29] and repeated measurements over time of such oxidized lipid products should be carried out to reinforce and validate for their role as biomarkers. Such assessment so far has been executed in Rett (RTT) syndrome where marked increased levels of isoprostanoids have been detected in typical RTT at every clinical stages of the disease [52].

## 4. Mechanisms Underlying Different Brain Diseases: Similar but Not the Same

Isoprostanoids, mainly F_2_-IsoPs, have been detected to be elevated in different neurological diseases from distinct etiological causes [36,53,54,55]. Due to the broad and general understanding of the isoprostanoids, they are repeatedly neglected for its specificity and often considered nonspecific indicators of oxidative damage. Nevertheless, F_2_-IsoP formation appeared to be modulated by specific mechanisms in the neurological diseases arising from methyl-CpG binding protein 2 (*MECP2*) gene expression, the so-called MECP2-pathies [56].

Isoprostanoid formation appears to be intimately linked to disorders of neurodevelopmental caused by alterations in the methyl-CpG binding protein 2 (*MECP2*) gene expression. In neurodevelopmental disorders, it is linked to under-or over-expression of *MECP2* gene, such as in RTT and *MECP2* duplication syndrome (MDS), where isoprostanoids formation have been shown to be specifically related to different *MECP2* gene mutations [25,26,52,57,58,59] (Table 1).

Moreover, in symptomatic RTT mice (*Mecp2* stop/y model), the amounts of 4-F_4t_-NeuroP and 10-F_4t_-NeuroP in brain tissue have been shown to be significantly higher than the wild-type, and highly correlated to the phenotypic severity [27].

RTT, which is due to de novo mutations in *MECP2* gene, features transient autistic-like phase [60] and has been recognized as model of neurodevelopmental disorders [61]. However, RTT is a progressive neurological disorder and not degenerative. Considering this, it is conceivable that various progressive neurological conditions, even on a degenerative basis, may share similar oxidative mechanisms. Usually, such common mechanisms involve the formation of isoprostanoids, albeit with their own specificity. Indeed, not only the products of the non-enzymatic lipid peroxidation but also the oxidation pathways were found to be relevant in previous studies (Table 1). By identifying the isomers that are synthesized in different oxidation pathways of the PUFA, one is able to (i) understand the time course of disease mechanisms, (ii) identify molecular targets (i.e., therapeutic target), and (iii) know the relationship with the clinical manifestations. Therefore, the availability of chemically synthesized molecules is crucial to test the clinical relevance of non-enzymatic oxidized products of PUFA.

## 5. In Search of a Biomarker: Isoprostanoids as Biomarkers in Neurological Diseases

Unlike other oxidative stress biomarkers, isoprostanoids provide unique information on the precursor and/or the targeted PUFA. Accordingly, isoprostanoid formation identifies which PUFA precursor is affected by the non-enzymatic oxidative process. Thus, F_2_-IsoP, F_4_-NeuroP, and F_2_-dihomo-IsoP levels are not only specific indices of oxidative stress but also markers of biological oxidative damage involving specific PUFAs.

According to the criteria for an ideal biomarker, it should be quantifiable to characterize a biological process and to predict clinical results, even after diet or drug intervention [62]. In particular, an ideal biomarker of oxidative damage (i) should detect a major part of total ongoing oxidative damage in vivo, (ii) should be measured employing robust technology, (iii) should not be confounded by diet, and (iv) should be stable on storage [63]. Indeed, several studies showed isoprostanoids do meet such criteria for neurological diseases [25,28,38,52]. Previous studies tested the non-enzymatic oxygenated metabolites of lipid peroxidation (i) in different pathologies [27,57,64], (ii) in groups of subjects at different ages [25], (iii) in relation to the severity of the disease [52,58], (iv) in relation to the pre-symptomatic clinical status of the disease [38], (v) in animal models [38], and (vi) in relation to drug treatment/supplementation [65].

In view of biomarkers as useful tools to identify targeted therapeutic treatment and drug development processes, knowledge of the molecular precursors of isoprostanoids has led to the experimentation of n-3 PUFAs supplementation in RTT. In subject treated with what specifically, a significant decrease in the non-enzymatic oxygenated metabolites of lipid peroxidation was observed, and a clinical improvement was found [52,59,65]. In these investigations [52,59,65], n-3 PUFA supplementation was not merely considered as a simple antioxidant treatment or a component in generic antioxidant defense; rather these studies interpreted n-3 PUFAs as biomolecules enriched in the brain lipids to prevent degeneration of the pathophysiological processes of the disease.

When considering the outcome of neural related diseases, it is crucial to consider the method of measurement as it needs to be sensitive enough for detection in order to avoid misinterpretation of data from rare samples. Currently, measurements of isoprostanoids in biological samples are widely carried out by mass spectrometry (MS), singly or tandem (MS/MS) such as gas chromatography—mass spectrometry (GC-MS), GC-MS/MS, liquid chromatography–mass spectrometry (LC-MS), and LC-MS/MS [66]. These accredited techniques for isoprostanoid determination in biological samples have advantages and disadvantages. Although identifications of the investigated molecules are specific and sensitive at low quantities (i.e., in the order of the picograms), it can be costly and time consuming. Furthermore, isoprostanoids are composed of numerous types of isomers due to hydrogen abstraction and molecular oxygen addition in the PUFA oxidation process, therefore it is necessary to unravel the molecular rearrangement of oxidized PUFA products mainly derived from AA, DHA, and AdA [31,67,68] that are richly found in the neurons.

The available scientific literature on F_4_-NeuroP and F_2_-dihomo-IsoP remains limited. This is mainly due to the limited availability of purified molecules to be used as reference compounds in the identification of isoprostanoids in biological samples, which is an indispensable step in the exploration of the cause-effect relationship between the neurological damage and the levels of isoprostanoids in the bloodstream or in other fluids and tissues. Nevertheless, assays performed in plasma or urine samples are proving useful to predict clinical presentation/evolution of neurological diseases [25,27,31,32,33,34,38,52,57,59,69,70] (Table 2). Plasma 10-F_4t_-NeuroP and 4-F_4t_-NeuroP levels were shown to be useful to discriminate between different brain diseases and the association to clinical severity appeared to be distinctive for different neurological conditions, thus suggesting that in vivo DHA oxidation follows preferential chemical rearrangements according to different human brain diseases. Consequently, the abundance of plasma 4-F_4t_-NeuroP and 10-F_4t_-NeuroP is able to predict disease severity (Table 3).

## 6. Conclusions and Future Research

**Figure 3:**
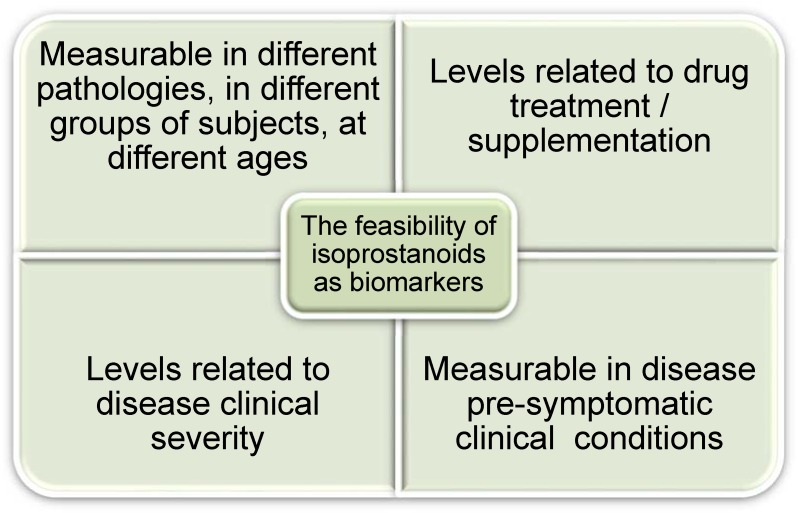
Characteristics that make isoprostanoids potentially useful biomarkers.

Although they are known about a quarter of century ago, the knowledge on the key role of isoprostanoids in the pathogenesis of experimental model and human diseases remain limited. Here, we summarized the current evidence on the role of isoprostanoids in the clinical presentation and evolution of several neurological diseases, ranging from very rare brain diseases to major health conditions taking into the account of worldwide socioeconomic impact (Figure 3). The current challenge is to identify the preferential biochemical pathways that actually follow oxidative reactions in the biological systems and, consequently, the identification of the specific isoprostane isomers related to the underlying pathogenic mechanism. It is clear that the accurate knowledge on the biological relevance of these molecules is the critical step that could be translated as technical/industrial advances of reliable biological disease markers and potentially aid in predicting the clinical progression and deciphering in the oxidative stress puzzle related to neurological disorders.

## Figures and Tables

**Table 1:** Isoprostanoids formation is related to altered *MECP2* gene expression.

Neurodevelopmental Disorders Caused by Alteration in the *MECP2* Gene Expression	Isoprostanoids		
	Plasma free F_2_-IsoPs	Plasma free F_4_-NeuroPs	Plasma free F_2_-dihomoIsoPs
Rett Syndrome (RTT)	+ ^a^	+ ^b^	+ ^c^
Most frequent *MECP2* mutations in RTT:			
R106W	↔ ^b^	↔ ^b^	
R 133C	↔ ^b^	↔ ^b^	
T158M	+++ ^b^	↔ ^b^	
R168X	+++ ^b^	+++ ^b^	
R255X	+++ ^b^	+++ ^b^	
R270X	+++ ^b^	++ ^b^	
R294X	↔ ^b^	↔ ^b^	
R306X	↔ ^b^	↔ ^b^	
C-terminal deletions	↔ ^b^	↔ ^b^	
Large deletions	↔ ^b^	++ ^b^	
*MECP2* duplication syndrome (MDS)	++ ^d^	++ ^d^	↔ ^d^

Legend: ↔, not significantly different as compared to control subjects; +, ++ and +++, increased highly increased and very highly increased, respectively, as compared to control subjects; C TER D, C terminal deletion; L DEL, large *MECP2* (methyl-CpG binding protein 2) gene deletion. ^a^ Leoncini et al. (2011) [58]; Signorini et al. (2014) [59]; De Felice et al. (2009) [26]; ^b^ Signorini et al. (2011) [52]; ^c^ De Felice et al. (2011) [25]; ^d^ Signorini et al. (2016) [57].

**Table 2:** F_4_-NeuroP and F_2_-dihomo-IsoP detection in different human and experimental brain disease models.

Human and Experimental Brain Disease Models	Sample	PUFA Non-Enzymatic Oxidized Products	Methodology	References
**Human Brain Disease**				
Alzheimer’s Disease	urine	F_2_-IsoPs, F_2_-dihomo-IsoPs, F_4_-NeuroPs	LC–MS/MS	García-Blanco et al. (2018) [32]
	brain	F_2_-IsoPs, F_2_-dihomo-IsoPs, F_4_-NeuroPs	GC-MS	VanRollins et al. (2008) [31]
Multiple sclerosis, Autism spectrum disorders, Rett syndrome, Down syndrome	plasma	4(RS)-4-F_4t_-NeuroP and 10(RS)-10-F_4t_-NeuroP	GC-MS/MS	Signorini et al. (2018) [27]
Down syndrome	plasma	F_2_-IsoPs, F_2_-dihomo-IsoPs, F_4_-NeuroPs	GC-MS/MS	Manna et al. (2016) [33]
Epilepsy	urine	4(RS)-4-F_4t_-NeuroP, 10-epi-10-F_4t_-NeuroP, 17-epi-17-F_2t_-dihomo-IsoP, 17-F_2t_-dihomo-IsoP, Ent-7(RS)-7-F_2t_-dihomo-IsoP, Ent-7-epi-7-F_2t_-dihomo-IsoP	HPLC-MS/MS	Medina et al. (2015) [34]
Traumatic brain injury	cerebrospinal fluid	Isofurans, F_4_-NeuroPs, F_2_-IsoPs	GC-MS	Corcoran et al. (2011) [70]
Rett syndrome	plasma	F_4_-NeuroPs ent-7(RS)-F_2t_-dihomo-IsoPs, 17-F_2t_-dihomo-IsoPs.	GC-MS/MS	Signorini et al. (2011) [52] Signorini et al. (2014) [59] De Felice et al. (2011) [25]
*MECP2* duplication syndrome	plasma	F_4_-NeuroPs	GC-MS/MS	Signorini et al. (2016) [57]
**Experimental Brain Disease**				
Perinatal hypoxic-ischemic damage	brain	F_4_-NeuroPs, neurofurans, F_2_-dihomo-IsoPs	LC–MS	Solberg et al. (2017) [69]
Rett syndrome	plasma brain	4(RS)-4-F_4t_-NeuroP and 10(RS)-10-F_4t_-NeuroP F_4_-NeuroPs, F_2_-IsoPs	GC-MS/MS	Signorini et al. (2018) [27] De Felice et al. (2014) [38]

Legend: F_2_-IsoPs, F_2_-isoprostanes; F_4_-NeuroPs, F_4_-neuroprostanes; F_2_-dihomo-IsoPs, F_2_-dihomo-isoprostanes; GC-MS, gas chromatography-mass spectrometry; GC-MS/MS, gas chromatography–tandem mass spectrometry; LC-MS/MS, liquid chromatography-tandem mass spectrometry; PUFA: polyunsaturated fatty acid.

**Table 3:** Plasma 4(RS)-4-F_4t_-NeuroP and 10(RS)-10-F_4t_-NeuroP abundancy and disease severity.

Human Disease	F_4_-NeuroPs		Plasma F_4_-NeuroP Levels Are Related to
	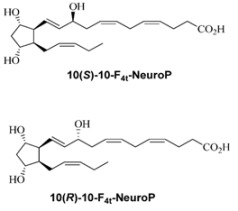	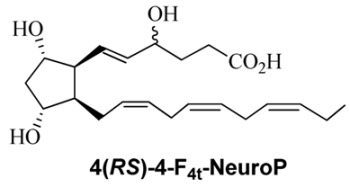	
Down syndrome	+ ^a^	**↔** ^a^	D I S E A S E S E V E R I T Y
Autism spectrum disorders	+ ^a^	**↔** ^a^	
Rett syndrome	+++ ^a^	++ ^a^	
Multiple Sclerosis	+++ ^a^	++++ ^a^	

Legend: ↔, not significantly different as compared to control subjects; +, ++, +++, and ++++, increased, highly increased, very highly increased, and extremely increased respectively, as compared to control subjects. ^a^ Signorini et al. (2018) [27].
